# Psychometric properties of the Canadian occupational performance measure in older individuals

**DOI:** 10.55730/1300-0144.5796

**Published:** 2023-11-28

**Authors:** Onur ALTUNTAŞ, Medine Nur ÖZATA DEĞERLİ, Ege TEMİZKAN, Gamze EKİCİ

**Affiliations:** Department of Occupational Therapy, Faculty of Health Sciences, Hacettepe University, Ankara, Turkiye

**Keywords:** Occupational performance, older people, reliability, validity

## Abstract

**Background/aim:**

The Canadian Occupational Performance Measure (COPM) is a person-centered assessment tool frequently used to identify occupational problems in older individuals and establish goals for geriatric rehabilitation. This study aims to assess the validity and reliability of the Turkish version of COPM (COPM-TR) in older people.

**Materials and methods:**

One hundred older people completed the COPM-TR, and 25 of them participated in a retest within two weeks. The convergent construct validity analyses of the COPM-TR included conducting correlation analyses between the COPM-TR and the Functional Independence Measure (FIM) and Lawton Brody Instrumental Activities of Daily Living (Lawton-IADL) scales. The Performance and Satisfaction subscales were subjected to an item analysis for the internal consistency of the COPM-TR. A test-retest analysis was conducted to investigate the reliability.

**Results:**

According to convergent construct validity analysis, it was found that there is a moderate correlation between COPM-TR performance and FIM (r = 0.51), and a strong correlation between Lawton IADL (r = 0.62). Similarly, there was a strong correlation between COPM-TR satisfaction and FIM (r = 0.61) and Lawton IADL (r = 0.61). Test-retest reliability was excellent for performance score and good for satisfaction score (ICC values 0.92; 0.78, respectively). COPM-TR showed excellent-high internal consistency (Cronbach’s alpha 0.92 for performance and 0.88 for satisfaction).

**Conclusion:**

COPM-TR is a valid and reliable tool that can be used to assess occupational performance in older people.

## 1. Introduction

The Canadian Model of Occupational Performance and Engagement (CMOP-E) is a conceptual model of occupational therapy that focuses on “occupational performance”, which constitutes the main goal of occupational therapists’ interventions [1–5]. According to the model, occupational performance is a person’s ability to select, organize, and perform meaningful, culture-appropriate, and age-appropriate occupations for self-care, enjoyment of life, and participation in social and economic life [6,7]. Occupational performance requires the dynamic interaction of the person, the environment and the professions throughout life, and these change with aging [8–10]. Older people have difficulties in the most familiar and simple occupations and consequently changes in occupational performance [11, 12].

Many older people have difficulty living independently in daily life [13]. Occupational therapists have a significant role in increasing the independence of the older people by identifying their occupational needs, ensuring their development, preventing disability and improving their quality of life [14–17]. Occupational therapists should conduct comprehensive assessments before making interventions for occupational performance [18]. They should use client-centered assessments to increase the motivation of their clients by actively involving them in the assessment process, taking into account their experiences, interests, and needs [19–21].

The Canadian Occupational Performance Measure (COPM) is a client-centered assessment designed to determine performance and satisfaction with the performance of the occupations that individuals experience in their daily lives and to determine priorities for intervention [22]. COPM is based on a semistructured interview between the client and the occupational therapist to identify important self-care, leisure, and productivity occupations that the client wants or is expected to do in daily life but cannot achieve [23]. Occupational therapists use the COPM to identify problem areas related to occupational performance, prioritize treatment and examine the effectiveness of treatment over time [24]. The five occupations that the client wants to prioritize in the intervention are identified. The client rates these five occupations from 1 (lowest) to 10 (highest) in performance and satisfaction [20]. The COPM has more than 25 years of history, and validity and reliability studies have been conducted in 36 languages [25]. It has Turkish validity and reliability in individuals diagnosed with multiple sclerosis [26]. Bianchini et al. stated that cultural adaptation of the COPM in different languages and populations is necessary for clinical practice and international comparability of this practice [27].

There is limited evidence regarding the use of COPM in the older population despite the increasing aging population worldwide and the occupational performance issues experienced by the older people [28, 29]. In older people, factors such as comorbidity, frailty, and cognitive impairment may affect the usability, validity, and reliability of the COPM [29]. Capdevila et al. stated that COPM might be an important concept for dealing with problems arising from aging in many countries [30]. In addition, the validity and reliability of assessments such as COPM should be performed because they support the effectiveness of occupational therapy within the health system [31, 32].

Since previous studies have emphasized the importance of COPM in geriatric rehabilitation and considering the increasing older population in Türkiye, this study aimed to determine the validity, reliability study and psychometric properties of the Turkish version of COPM (COPM-TR), an occupational-based assessment, in older individuals.

## 2. Materials and methods

### 2.1. Study design and ethical issues

This study was designed as a methodological validity and reliability study. The study was conducted by the Declaration of Helsinki and approved by the Hacettepe Non-Interventional Ethics Committee with decision number 2023-04/41 and research number GO 23/01. Verbal and written informed consent was obtained from the participants before the study.

### 2.2. Participants

The study included older individuals at the Hacettepe University Geriatric Rehabilitation Unit. The sample size was 100 participants for validity and reliability studies [33]. Inclusion criteria were as follows: (I) being 65 years of age or older, (II) scoring 24 or above on the Mini-Mental State Examination Test [34], and (III) having no neurologic or psychiatric diagnosis that could affect the assessment. Individuals with hearing and/or visual impairment that might interfere with the assessments were excluded. A total of 127 older individuals were evaluated; participants who did not want to continue the study (7), visual and/or hearing impairment (11), and at risk in terms of cognitive status (9) were excluded.

### 2.3. Data collection tools

Participants completed the demographic information form, Functional Independence Scale (FIM), Lawton & Brody Instrumental Activities of Daily Living Scale (Lawton-IADL), and COPM-TR. One-fourth of the participants (n = 25) had the COPM-TR repeated two weeks after the first evaluation.

#### 2.3.1. Demographic information form

To determine the general status of the participants, a demographic information form that evaluated age, sex, marital status, educational status, chronic disease, and drug and smoking use was used.

#### 2.3.2. Functional Independence Scale (FIM)

FIM was used to examine convergent construct validity of the COPM-TR. FIM was developed to assess the independence of individuals in their basic physical and cognitive activities in daily life [35]. It consists of 18 questions, including motor function (13 items) and cognitive function (5 items). Each item is scored 1 (dependent) −7 (independent). The total score is between 18–126; the higher the score, the higher the independence level of the individual. The Turkish validity and reliability study of the scale was conducted by Küçükdeveci et al. The Cronbach’s alpha coefficient was 0.957 for motor function and 0.922 for cognitive function [36].

#### 2.3.3. Lawton & Brody Instrumental Activities of Daily Living Scale (Lawton-IADL)

The scale was used to assess individuals’ daily living activities. It consists of 8 questions that include information about use phone, meal preparation, shopping, daily household chores, laundry, travel, medication use, and financial affairs [37]. Each question is scored between 0–3 (3–completely independent and 0–dependent). A total score ranging from 0–8 points is categorized as dependent, 9–16 points as semidependent, and 17–24 points as independent. The validity and reliability of the scale were performed by Güzel et al.; the Cronbach’s alpha value is 0.85 [38].

#### 2.3.4. Turkish version of COPM (COPM-TR)

Law et al. designed the COPM, a person-centered assessment so that occupational therapists to evaluate their clients with a focus on occupational performance [39]. The COPM is based on a semistructured interview between the client and the occupational therapist and involves self-assessment by the client of occupational performance in three performance domains: self-care, productivity, and leisure [40]. The COPM application consists of four steps: (i) the client identifies the occupations they have difficulty within the areas of self-care, productivity, and leisure; (ii) the client rates the importance of these occupations from 1 (least important) to 10 (most important); (iii) the client is asked to rate the five occupations they consider most important in terms of performance and satisfaction on a scale of 1 (1: not able to do and not satisfied at all) to 10 (10: doing extremely well and extremely satisfied) [41]. Performance and satisfaction scores are determined by summing the obtained performance and satisfaction scores and dividing by the number of occupations [42]. The Turkish validity and reliability of the scale in individuals diagnosed with multiple sclerosis was conducted by Torpil et al. The Cronbach’s alpha value of the scale is 0.92 [26].

### 2.4. Statistical analysis

All statistical analyses were conducted using SPSS v26 software, while post-hoc power analyses were conducted using G*Power 3.1.9.7 and PASS 2023 software. All variables’ distribution was compared against the normal distribution using Kolmogorov-Smirnov test. To analyze the central tendency of both the continuous demographic variables and assessment results, mean and standard deviations were calculated. For investigating the dispersion of discrete demographic variables as well as the discrete assessment results, frequencies were calculated along with the percentages.

Prior to conducting validity and reliability analyses, all participants’ recorded activities in the COPM-TR interviews were analyzed for understanding the commonly reported activities and activity selection trends within the sample. Results from this analysis were reported using frequency and percentage values. In all analyses, the significance level was considered to be 95% (p < 0.05). The COSMIN (Consensus-based standards for the selection of health status measurement instruments) guidelines were followed for the validity and reliability analyses [43].

The validity of the COPM-TR was investigated using convergent and divergent construct validity. For the convergent construct validity, the “Hypothesis Testing for Construct Validity” method defined within the COSMIN guidelines was utilized. As per this methodology, two closely related assessments that are valid and reliable in Turkish were selected; FIM and Lawton-IADL. The correlations between the COPM-TR and the two scales wereanalyzed using the Spearman Correlation Coefficient. The interpretation of the correlation coefficient was done as; r < 0.30: “weak correlation”, r = 0.31–0.60: “moderate correlation”, r = 0.61–0.90: “strong correlation” and r > 0.90: “excellent correlation” [44]. All three scales are aimed at occupational performance, but the COPM is applied using an interview, while the FIM and the Lawton-IADL utilizes a patient-reported survey structure. There are also differences in the scope of the three scales in terms of the types of ADL they focus on. Therefore, it was hypothesized that there would be a low to moderate relationship between COPM-TR and FIM, as well as between COPM-TR and Lawton-IADL. The divergent construct validity was investigated by comparing the COPM-TR scores between sexes. As the COPM-TR does not intrinsically yield different or distinctive results based on sexes, no significant difference was expected to be found.

For the reliability of the COPM-TR, the test-retest reliability was analyzed, and an item analysis was performed. For the test-retest reliability, the interclass correlation coefficients (ICC) were calculated for both the performance and satisfaction subscores of the COPM-TR. The ICC values were interpreted as; ICC = 0–0.49: “weak reliability”, ICC = 0.50–0.74: “moderate reliability”, ICC = 0.75–0.89: “good reliability” and ICC = 0.90–1: “excellent reliability” [45]. The item analysis was conducted in both the performance and the satisfaction subscores for understanding the internal consistency of the COPM. The interpretation of the internal consistency was done using the Cronbach’s alpha value as; <0.60: “Not acceptable”, 0.60–0.79: “Acceptable”, 0.80–0.89: “High” and >0.89: “Excellent” [46].

## 3. Results

A total of 100 participants were included in the analysis. The participant’s mean age was 70.84 ± 6.67, 28 participants were male (28%) and 72 participants were female (72%). Other demographic information of the participants is presented in Table 1.

The participants’ activity selections were investigated, and it was found that the participants most commonly reported household management and self-care activities such as cleaning, shopping and taking a bath/shower for their 1st activity and added recreational activities such as gardening and socializing in their 2nd, 3^rd^, and 4th activities. The reported activities are presented in detail in Table 2 and Table 3.

The convergent construct validity analyses of the COPM included conducting correlation analyses between the COPM and the FIM and Lawton-IADL scales. The analyses showed that, as hyphothesized, both COPM subscales showed weak-to-moderate correlations with the FIM and Lawton-IADL scales (Table 4). Post-hoc power analyses were conducted for the correlation analyses between the COPM and the FIM and Lowton-IADL scales. The power analyses showed the lowest achieved power size among all analyses as 92% when calculated by the lowest correlation coefficient of 0.33, the sample size of 100 and alpha error probability of 5%.

The divergent construct validity analysis showed that there were no significant differences between sexes in COPM’s performance and satisfaction scales (Table 5).

A test-retest analysis was conducted for investigating the reliability of the COPM. A two-way random effects model with absolute agreement was utilized for the analysis and the results yielded an ICC score of 0.92 (Excellent reliability) for COPM’s Performance subscale and an ICC score of 0.78 (Good reliability) for COPM’s satisfaction subscale. The test-retest analysis is presented in Table 6. Post-hoc power analyses were conducted for both test-retest reliability analyses, using the hypothesis testing method. In the post-hoc power analyses, the null hypothesis was considered to be ICC = 0.6 because the consensus-based cut-off point for sufficient reliability is at an ICC value of 0.7, and an ICC value of 0.6 is considered insufficient [33]. Calculating for a null hypothesis ICC value of 0.6, an alpha error probability of 5%, and a sample size of 100, the achieved power sizes were 93.7% for COPM Satisfaction and 99.9% for COPM performance analyses.

In order to show the internal consistency of the COPM-TR, the performance and satisfaction subscales were subjected to an item analysis. The results showed a Cronbach’s alpha score of 0.92 (Excellent internal consistency) for the performance and 0.88 (high internal consistency) for the satisfaction subscale. The item analysis for the performance and satisfaction subscales are presented in Table 7 and Table 8 respectively.

## 4. Discussion

This study confirms that COPM-TR is a validate and reliable instrument for measuring occupational performance and satisfaction with performance for older people.

COPM is useful in the process of prioritizing problem areas and setting goals for response and program planning. Identified problems form the basis for determining targeted outcomes and setting priorities for intervention [47]. Similar to the studies conducted, we found that among all the problems defined [40, 48], the most frequently mentioned problems belonged to self-care activities. In the study examining COPM in 225 older individuals, it was stated that the participants’ self-care activity area and especially functional mobility were given priority in this area [49]. However, in our study, the fact that taking a bath/shower activity was more important for self-care activity and that mobility was ranked 5th as a problem area was a remarkable result. It has been observed that the activities for gardening and socialization are in the 2nd, 3^rd^, and 4th places, and that the participants especially think about the problems experienced in leisure activities in the background. It is thought that the minimum level of reporting of problems related to productive activities is due to the retirement of the participants.

Tuntland et al. examined the validity of COPM in Scandinavia for older people with functional decline who still live in their own homes and participate in multidisciplinary home-based rehabilitation for older people. In this study, it was reported that the content validity, construct validity, and feasibility of COPM were sufficient [49]. The convergent validity and predictive validity of COPM with patients treated in the subacute clinic were examined by Roe et al. In this study, it was stated that COPM was significantly associated with FIM and SF-36 [50]. Donnelly et al. conducted a study in which 41 participants with spinal cord injuries completed FIM and COPM. FIM Motor scale scores at discharge were significantly associated with COPM Satisfaction and Performance scale scores [51]. In the study by Chan and Lee, which examined the validity of COPM by associating it with FIM, 39 participants with orthopedic and stroke-related health problems completed both scales. It was stated that the relationship between COPM and FIM was significant [52]. In our study, the convergent construct validity analyses of the COPM included conducting correlation analyses between the COPM and the FIM and Lawton-IADL scales. The analyses showed that, both COPM subscales showed weak-to-moderate correlations with the FIM and Lawton-IADL scales.

In a study by Cup et al. on patients with stroke, test-retest reliability of the COPM was moderate for the item pool but was good for the performance and satisfaction scores [53]. Atashi Neda et al. in the Persian version study of COPM, they applied test-retest to older participants with an interval of one week and again found the level of reliability to be moderate [48]. In the Italian version study of COPM in individuals diagnosed with Parkinson’s by Bianchini et al., they performed item analysis with the test-retest method for reliability and found moderate reliability and good internal consistency [27]. Kjeken et al. performed test-retest two weeks later on participants with ankylosing spondylitis for reliability analysis in the Norwegian version of COPM and found good reliability [47]. Torpil et al. in the Turkish version study of COPM in individuals with multiple sclerosis, the reliability analysis was performed with the test-retest method at one week intervals and high reliability was found [26]. The fact that the COPM had excellent reliability and good internal consistency in our study is in line with the literature.

This study has several limitations. The fact that our study sampling method was not random and the majority of participants were female may have affected the research results. In addition, perhaps the most important limitations of this study is that the older people have difficulty in scoring the occupations. The difficulty experienced in scoring COPM-TR was that it was stated that it was enough to be able to do so much at this age, and therefore, high satisfaction points were given to the problems with low performance.

As a result, it has been found that COPM-TR, which reveals the occupational performance problems of the individual with the individual’s own perception, and rates the performance of the individual in the determined occupations with his own perception, and the satisfaction with his performance, is a valid and reliable measurement in older individuals. It is thought that COPM-TR will guide health professionals working in this field in determining the activity problems of older individuals in the areas of self-care, productivity and leisure and planning target-oriented interventions for these areas.

## Figures and Tables

**Table 1:** Participants’ demographic information.

Demographic information	Mean±SD	
Age	70.84±6.67	
**Demographic information**	**n**	**%**
**Sex**		
Male	28	28%
Female	72	72%
**Marital status**		
Single	34	34%
Married	66	66%
**Education**		
Illiterate	23	23%
Primary school	46	46%
Secondary school	12	12%
High school	8	8%
University	11	11%
**Has a chronic disease**		
Yes	79	79%
No	21	21%
**Routinely uses medication**		
Yes	76	76%
No	24	24%
**Smoking**		
Yes	18	18%
No	82	82%

**Table 2:** Participants’ reported activities in the COPM-TR interviews.

	n (%)
**1** **^st^** ** Activity**	
Cleaning	12(12%)
Shopping	11(11%)
Taking a bath/shower	9(9%)
Using a phone	9(9%)
Cooking	7(7%)
Knitting	7(7%)
Using the toilet	6(6%)
Reading	5(5%)
Performing prayer	5(5%)
Other	29(29%)
**2** **^nd^** ** Activity**	
Did not report an activity	1(1%)
Gardening	14(14%)
Cooking	13(13%)
Taking a bath/shower	11(11%)
Cleaning	10(10%)
Socializing	8(8%)
Climbing up and down stairs	6(6%)
Doing sports	5(5%)
Withdrawing money from an ATM	4(4%)
Reading	4(4%)
Other	24(24%)
**3** **^rd^** ** Activity**	
Did not report an activity	7(7%)
Cleaning	11(11%)
Socializing	10(10%)
Cooking	8(8%)
Shopping	7(7%)
Using public transport	5(5%)
Gardening	4(4%)
Knitting	4(4%)
Spending time with grandchildren	4(4%)
Dressing	4(4%)
Other	36(36%)
**4** **^th^** ** Activity**	
Did not report and activity	34(34%)
Cooking	6(6%)
Cleaning	6(6%)
Using a phone	5(5%)
Knitting	5(5%)
Socializing	5(5%)
Gardening	4(4%)
Using public transport	4(4%)
Other	31(31%)
**5** **^th^** ** Activity**	
Did not report and activity	70(70%)
Doing sports	6(6%)
Using public transport	4(4%)
Spending time with grandchildren	3(3%)
Withdrawing money from an ATM	2(2%)
Gardening	2(2%)
Cleaning	2(2%)
Reading	2(2%)
Going on a walk	2(2%)
Other	7(7%)

**Table 3:** Participants’ reported areas of activities of daily living in the COPM-TR interviews.

	n(%)
**1** **^st^** ** activity**	
Self-care	44(44%)
Productivity	24(24%)
Recreation	32(32%)
**2** **^nd^** ** activity**	
Did not report an activity	1(1%)
Self-care	41(41%)
Productivity	24(24%)
Recreation	34(34%)
**3** **^rd^** ** activity**	
Did not report an activity	7(7%)
Self-care	37(37%)
Productivity	21(21%)
Recreation	35(35%)
**4** **^th^** ** activity**	
Did not report an activity	34(34%)
Self-care	29(29%)
Productivity	11(11%)
Recreation	26(26%)
**5** **^th^** ** activity**	
Did not report an activity	70(70%)
Self-care	11(11%)
Productivity	5(5%)
Recreation	14(14%)

**Table 4:** Correlations between COPM-TR, FIM and Lawton-IADL scales.

COPM subscale		FIM Motor	FIM Cognitive	FIM Total	Lawton-IADL
**COPM performance**	r	0.54	0.33	0.51	0.62
**COPM satisfaction**	r	0.55	0.48	0.61	0.61

**Table 5:** Differences between sex COPM’s performance and satisfaction subscales.

	Male X ± SD	Female X ± SD	p
**COPM performance**	4.93 ± 1.81	5.54 ± 1.77	0.09
**COPM satisfaction**	5.10 ± 1.83	5.36 ± 1.78	0.46

**Table 6:** Test-retest analysis for the COPM-TR.

	First assessment X ± SD	Second assessment X ± SD	ICC	95% Confidence interval
**COPM performance**	5.37 ± 1.79	4.80 ± 1.44	0.92	0.81–0.96
**COPM satisfaction**	5.28 ± 1.79	4.81 ± 1.42	0.78	0.50–0.90

**Table 7:** Item analysis of the COPM’s performance subscale.

COPM Performance subscale item	Scale mean if item deleted	Scale variance if item deleted	Corrected item-total correlation	Squared multiple correlation	Cronbach’s alpha if item deleted
**Item 1**	21.43	48.87	0.79	0.68	0.89
**Item 2**	22.00	47.86	0.83	0.70	0.88
**Item 3**	21.13	49.29	0.85	0.74	0.88
**Item 4**	21.70	42.83	0.76	0.63	0.91
**Item 5**	21.60	49.35	0.73	0.56	0.90

**Table 8:** Item analysis of the COPM’s satisfaction subscale.

COPM satisfaction subscale item	Scale mean if item deleted	Scale variance if item deleted	Corrected item-total correlation	Squared multiple correlation	Cronbach’s alpha if item deleted
**Item 1**	21.73	46.61	0.72	0.63	0.84
**Item 2**	21.93	47.37	0.70	0.63	0.84
**Item 3**	21.23	50.25	0.80	0.69	0.83
**Item 4**	21.73	46.96	0.65	0.57	0.86
**Item 5**	21.10	49.19	0.65	0.54	0.85
